# P02-010 - A novel 24 nucleotide deletion in the TNFRSF1A

**DOI:** 10.1186/1546-0096-11-S1-A117

**Published:** 2013-11-08

**Authors:** DM Rowczenio, H Trojer, G Wang, PN Hawkins, HJ Lachmann, NM Stewart

**Affiliations:** 1 Medicine, University College London, London, UK

## Introduction

TNF receptor associated periodic syndrome (TRAPS) is a rare autosomal dominant disease characterized by episodes of fever usually lasting two to three weeks, associated with severe abdominal pain, arthralgia, skin rash and red and swollen eyes. The age of onset varies from early childhood to adulthood, and the disease affects both sexes equally. Although first cases were described in individuals of Irish-Scottish descent, TRAPS has since been reported world-wide.

## Case report

A 41 year old British man, with no family history, presented in early adolescence with episodes of severe abdominal pain for which he had an appendectomy at the age of 12. He had 10 to 12 attacks per year each lasting almost exactly two weeks accompanied by a headache, abdominal pain, arthralgia, myalgia, night sweats, generalised erythema and unilateral non-painful cervical lymphadenopathy. He had occasional red eyes but no periorbital oedema or periorbital pain. His puberty was relatively late and he is significantly shorter than his siblings. During attacks his inflammatory markers, serum amyloid A protein (SAA) and C-reactive protein (CRP), were elevated to median values of 258 mg/L (range 60 – 679) and 46 mg/L (range 30 – 96) respectively. He is in full time employment and remains physically extremely fit.

He underwent screening of the four genes associated with periodic fever syndrome: *MEFV* (the gene associated with FMF); *TNFRSF1A* (the gene associated with TRAPS) *NLRP3* (the gene associated with CAPS) and *MVK* (the gene associated with MKD).

A novel in frame deletion of 24 nucleotides (c.255_278del) in exon 3 of the *TNFRSF1A* gene was identified by PCR and Sanger sequencing and subsequently confirmed with allele-specific PCR by using primers complementary to the sequence of a mutant DNA. Screening of parental DNA showed no evidence of nucleotide alternation in their *TNFRSF1A* gene suggesting that the deletion identified in our patient was a de novo mutation.

He commenced treatment with anakinra, to which had a dramatic response with a rapid resolution of symptoms and normalization in SAA and CRP to healthy levels of 8.1 and 4 mg/L.

## Discussion

Genetic aberrations in tumor necrosis factor receptor superfamily 1A gene (*TNFRSF1A*) located on chromosome 12p13 are the cause of TRAPS. To date, 89 variants have been reported to be associated with clinical TRAPS, of which 95% are single nucleotide substitutions.

The novel mutation identified in our patient results in a deletion of eight amino acids p.Ser86_Glu93del (Infever description S57_E64del) in the second domain of the tumor necrosis factor receptor superfamily member 1A protein (TNFR1).

Based on the crystal structure of TNFR1 proposed by Banner et al, residues from Ser57 to Phe60 are structurally conserved between the second and third extracellular domains. Additionally Phe60 residue is crucial for proper domain 2 folding and its side chain further stabilizes the structure by interacting with both the second disulfide bridge and the Ser-74-Asp-93 hydrogen bond bridge. It is therefore very likely that the novel variant we have identified produces profound changes to the three dimensional shape of the TNFR1 impairing its folding and binding with TNF.

## Disclosure of interest

None declared.

